# Recent improvement in operative techniques lead to lower pacemaker rate after Perceval implant

**DOI:** 10.1093/icvts/ivac182

**Published:** 2022-06-25

**Authors:** Olivier Fabre, Mihai Radutoiu, Ionut Carjaliu, Olivier Rebet, Laurence Gautier, Ilir Hysi

**Affiliations:** Department of Cardiac Surgery of Artois, Centre Hospitalier de Lens et Hôpital Privé de Bois Bernard, Ramsay, Santé, France; Department of Cardiac Surgery of Artois, Centre Hospitalier de Lens et Hôpital Privé de Bois Bernard, Ramsay, Santé, France; Department of Cardiac Surgery of Artois, Centre Hospitalier de Lens et Hôpital Privé de Bois Bernard, Ramsay, Santé, France; Department of Cardiac Surgery of Artois, Centre Hospitalier de Lens et Hôpital Privé de Bois Bernard, Ramsay, Santé, France; Department of Cardiac Surgery of Artois, Centre Hospitalier de Lens et Hôpital Privé de Bois Bernard, Ramsay, Santé, France; Department of Cardiac Surgery of Artois, Centre Hospitalier de Lens et Hôpital Privé de Bois Bernard, Ramsay, Santé, France

**Keywords:** Pacemaker rate, Perceval, Minimally invasive, Aortic valve replacement

## Abstract

**OBJECTIVES:**

Our goal was to compare pacemaker rate usage following two different operating techniques for implanting the Perceval aortic valve replacement.

**METHODS:**

In this retrospective, single-centre study, we studied patients with isolated or concomitant Perceval aortic valve replacement operated on first between April 2013 and January 2016, following traditional operating techniques, with patients operated on between January 2016 and December 2020, after the adoption of a modified protocol based on different annulus sizing, higher positioning of the valve and no ballooning after valve deployment was adopted. The operations were performed by 2 surgeons, and patients were followed-up for a period of 30 days.

**RESULTS:**

A total of 286 patients, with a mean age of 77 (4.9) years, had Perceval valves implanted during the study period, of which 79% were isolated aortic valve procedures. Most patients (66.8%) underwent minimally invasive procedures. Cross-clamp time was 55.1 (17.6) min. The overall postoperative pacemaker insertion rate was 8.4%, which decreased decisively after the 2016 change in the implant protocol (16% vs 5.6%; *P* = 0.005), adjusted odds ratio of 0.31 (95% confidence interval: 0.13–0.74, *P* = 0.012). Univariable and multivariable analysis showed that larger valve size (*P* = 0.01) and ballooning (*P* = 0.002) were associated with higher risk of implanting a pacemaker. Postoperative 30-day mortality was of 4.5%.

**CONCLUSIONS:**

Improvement in the operating techniques for implanting the Perceval valve may decrease the rate of pacemakers implanted postoperatively. Although further studies are needed to confirm these results, such a risk reduction may lead to wider use of Perceval valves in the future, potentially benefiting patients who are suitable candidates for minimally invasive surgery.

**Central picture.**
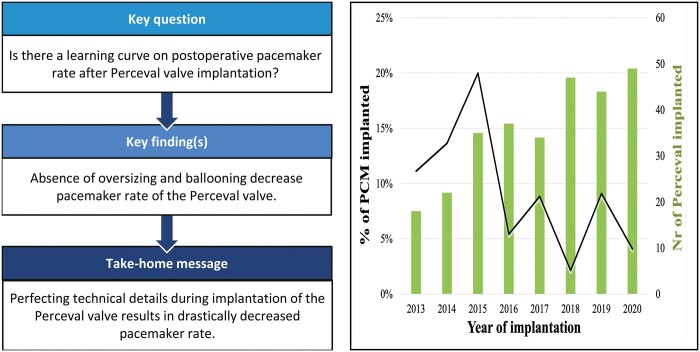
Decrease in the pacemaker rate after Perceval implants in the last 5 years. The left Y-axis shows the percent of permanent cardiac pacemakers implanted. The right Y-axis (in green) shows the number of Perceval valves implanted.

## INTRODUCTION

Implanting the sutureless nitinol Perceval valve (CORCYM, previously LivaNova, Saluggia, Italy) is a well-established aortic valve replacement procedure. Following the initial conceptual and pilot trial [1, 2], more than 200 publications on the Perceval valve are currently referenced in the published literature. This valve replacement modality was particularly useful in cases of minimally invasive surgery, especially a right anterior minithoracotomy [3], because it greatly facilitates the surgical procedure. Recently, results from the prospective randomized PERSIST trial [4] showed that the Perceval valve was not inferior to sutured valves, despite concerns about increased postoperative permanent pacemaker risk among its recipients.

The goal of the present study was to assess the potential role of technical adaptations and improvements in the operating protocol for the Perceval valve implants, by analysing series of patients operated on by 2 senior surgeons (OF, IH) and to discuss our experience of improving outcomes over the years.

## PATIENTS AND METHODS

### Patients

This is a retrospective, single-centre review of all patients who had Perceval S valves implanted in our department from April 2013 to December 2020. During the whole period of the study, only the Perceval S valve was implanted. We limited our study to patients who received an aortic valve replacement through a sternotomy or minimally invasive surgery and without involvement of aortic root procedures or aortic endocarditis. Preoperative rhythm disturbances (first degree atrioventricular block, left or right bundle branch block) were searched for on the medical charts of all patients. Although relatively rare, procedures performed in emergency settings were included in this study; cases with concomitant procedures of coronary artery bypass were also included in the study. Because we focused on pacemaker use after receiving a Perceval implant, we excluded 3 patients who needed early reoperations with secondary implants of a sutured valve. These 3 patients were operated on in the beginning of our experience with Perceval, and they presented with severe paravalvular regurgitation due to incomplete aortic annular debridement. None of these 3 had a pacemaker implanted postoperatively.

### Ethics statement

Due to the retrospective nature of the study, our institutional review board ruled that there was no need for informed patient consent (DELIBERE_CERC-SFCTCV-2022–02-01_18018).

### Surgical technique

The sternotomies were performed as usual with antegrade cold blood cardioplegia repeated every 30 min. A right anterior minithoracotomy or ministernotomy was also performed as described elsewhere [5, 6]. Briefly, a minithoracotomy was performed through an anterolateral incision at the level of the second or third intercostal space. The ministernotomy was either superior, inferior or inversed C-shaped. The superior ministernotomy was performed through an inverted L incision of the sternum from the manubrium to the level of the second or third intercostal space. The inferior ministernotomy was performed through an inverted L incision in the sternum from the xiphoid to the second intercostal space. Finally, the reversed C-shaped ministernotomy was performed through a double incision on the right side of the sternal body, between the second and the fourth intercostal spaces. In all patients, valve function was assessed through transoesophageal echocardiography before weaning from cardiopulmonary bypass.

Two different Perceval valve implant protocols were used at 2 distinct time periods within the time frame covered by this study. Between April 2013 and January 2016, Perceval valves were implanted according to the manufacturer’s instructions, after removal of the native aortic valve and subtotal decalcification of the annulus. Any aortic annulus damage at this stage was repaired with a 5/0 polypropylene suture before implanting the Perceval valve. The correct valve size was selected by measuring the white part of the obturator (valve sizer) that would not pass through the annulus. The 3 guiding sutures were placed 2 mm below the aortic annulus, at the nadir of each cusp and finally, the valve was ballooned at 4 atm for 30 s.

**Figure 1:**
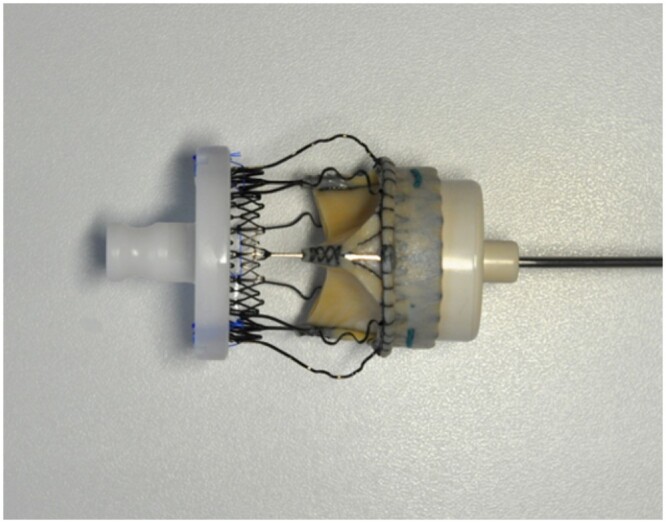
The outer diameter of the obturator (sizer) of the Perceval is always smaller than the inner diameter of the related valve.

An interim analysis of results performed during January 2016 identified a high postoperative pacemaker rate and led to the decision to modify our implant technique. Firstly, total decalcification of the aortic annulus was done with meticulous debridement, as we routinely did for sutured valves. Secondly, because the inner diameter of the inflow portion of the stent of the Perceval valve appeared to be larger than the outer diameter of the corresponding white part of the obturator (Fig. 1), we chose the size of the valves in which the white obturator remained stable above the aortic annulus but could pass through the annulus itself while pushed only with a gentile force (this is due to the elasticity of the aortic annulus). If you had to push with a significant force in order to pass the annulus or if the annulus could not be passed with the white obturator, we chose the lower size valve. In addition, the guiding sutures were no longer passed below the annulus but instead through the aortic annulus itself, which resulted in a higher position of the valve. Finally, we also stopped the practice of ballooning at the end of the implant procedure. Identical techniques and changes were simultaneously adopted by both surgeons, whose interventions are reviewed in this work.

### Postoperative outcomes

Postoperatively, the patients were moved to dedicated intensive care units and then to the wards. They were subsequently referred to rehabilitation centres or secondary cardiology departments or were fully discharged. Information on a 30-day follow-up period was collected through telephone interviews with the patients’ cardiologists or referring physicians. The follow-up of the cohort was 100% complete.

Hospital morbidity and mortality data were collected over a period of 30 days. Postoperative mortality was defined as death within 30 days of surgery, whether in-hospital or after discharge. Postoperative morbidity included every medical complication at any time after the operation that prolonged the stay in the intensive care unit or ward for more than 2 days or 5 days, respectively. Pacemaker usage was defined as an implant performed within 30 days from the intervention. It is important to underline that no changes in pacemaker implant recommendations were made over the entire period covered by this study. The management of patients with postoperative high conductance disorders (complete atrioventricular blocs and not-isolated left or right branch bundle blocs) was decided jointly with the electrophysiology cardiologists in our department. Efforts were made to avoid pacemaker implants before postoperative day 4.

We also assessed the postoperative length of stay, mean gradient and the Vmax of the valve by viewing the discharge transthoracic echocardiography scans. Also, because concerns have been raised about postoperative thrombopenia after a Perceval implant [4], we decided to report our data on this subject as a secondary end point. Thrombopenia was defined as a fall in platelet counts below 150 000 10^9^/l and was deemed severe at levels below 50 000 10^9^/l.

### Statistical analyses

Statistical analyses were performed using SEM (Statistique Epidémiologie Médicine) software, Clermont-Ferrand, France. The cohort was separated into 2 groups according to the technique used to implant the valve and its modification in January 2016. Mean values and standard deviations, as well as medians with interquartile ranges, are used to describe the distribution of continuous variables, whereas percentages are used for categorical variables. Normality of distribution was evaluated using the Shapiro–Wilk test. Characteristics of subjects were compared using the two-tailed χ^2^ or the Fisher test for categorical variables and the Student *t*-tests for continuous variables. In case of non-normal distribution of continuous variables, the Mann–Whitney *U* test was used. A Bonferroni correction was applied in the subgroup analysis of pacemakers implanted in patients with isolated aortic valve replacements. Clinically relevant variables, potentially affecting the risk of postoperative pacemaker implants, were assessed in univariable analysis separately in the 2 groups. The variables that were significantly associated with the rate of pacemakers implanted postoperatively and the variables that were not equally distributed between the 2 groups (age, gender, combined surgery, bicuspid aortic valve, ministernotomy, valve size and ballooning), were subsequently included in a stepwise forward-selection multivariable Cox model. The adjusted odds ratio for the risk of implanting a pacemaker was calculated between the 2 different periods.

## RESULTS

### Patient baseline characteristics

During the 8-year period covered by this study, a total of 286 patients were operated on for aortic valve replacement with Perceval implants at our centre. Table 1 summarizes patient baseline characteristics. The mean age of the entire cohort was 77 (4.9) years; most patients (51.6%) were women. Patients were younger in the second part of the study (76.2 vs 78.9 years; *P* = 0.0006) in accordance with the development of indications for transcatheter aortic valve replacement in older patients. Women were more frequently operated on before 2016 (72% vs 44.3%; *P* = 0.0003). Isolated aortic stenosis was the most common underlying reason for surgery (79%) and was more frequent in the first part of the study (88% vs 75.8%; *P* = 0.02). Combined surgery was performed in 60 patients, most of whom (54 patients) had coronary artery bypass surgery. In the 6 remaining patients, the concomitant procedures were distributed as follows: 2 maze procedures, 2 tricuspid annuloplasties, 1 Morrow septal myectomy and 1 ventricular septal defect closure. Urgent interventions and reoperations were rare (1.7% and 4.2%, respectively). Preoperative electrocardiographic rhythm disturbances did not differ between the 2 cohorts (*P* = 0.31). The average EuroSCORE II was not statistically different between the 2 periods of the study (2.6 vs 2.2; *P* = 0.06).

**Table 1:** Baseline characteristics of the patients before and after 2016

Total patients = 286:	75 patients (< 2016), mean (SD)/% (n)	211 patients (> 2016), mean (SD)/% (n)	*P*-Value
Age	78.9 (5.5) years	76.2 (4.4) years	0.0006
Women	72% (54)	44.3% (93)	0.0003
BMI	27.7 (4.7) kg/m²	28.8 (5.1) kg/m²	0.08
EF	58.7 (7.7) %	59.1 (8.3) %	0.51
Current smokers	8% (6)	10.4% (22)	0.65
Hypertension	88% (66)	89.1% (188)	0.80
Dyslipidaemia	44% (33)	41.7% (88)	0.73
Diabetes mellitus	26.6% (20)	36.9% (78)	0.11
PAD	17.3% (13)	12.8% (27)	0.33
Anticoagulated atrial fibrillation	21.3% (16)	23.2% (49)	0.74
COPD	5.3% (4)	6.6% (14)	0.90
Ischaemic cardiomyopathy	18.6% (14)	29.8% (63)	0.06
Emergency surgery	1.3% (1)	1.9% (4)	0.85
Preoperative ECG rhythm disturbances	13.3% (10)	8.5% (18)	0.31
Redo	6.62% (5)	3.3.2% (7)	0.36
Isolated AVR	88% (66)	75.8% (160)	0.02
Combined surgery	12% (9)	24.2% (51)	0.03
• CABG	7	47	
• Other	2	4	
EuroSCORE II	2.6 (1.7)	2.2 (1.6)	0.06

AVR: aortic valve replacement; BMI: body mass index; CABG: coronary artery bypass grafting; COPD: chronic obstructive pulmonary disease; ECG: electrocardiogram; EF: ejection fraction; PAD: peripheral artery disease; SD: standard deviation.

### Intraoperative characteristics

The main intraoperative observations are summarized in Table 2. Most of the patients underwent a minimally invasive approach (66.8%, n = 191), with the right anterior minithoracotomy being the most commonly performed procedure (n = 104, 54.4%). Bicuspid valves were uncommon for the entire cohort (12.2%), always of type I and more frequent in the second part of the study (5.3% vs 14.7%, *P* =  0.03), presumably because Perceval valve implants were initially contraindicated in these patients and because the age of the cohort was younger in the second part of the study. Cross-clamp times were lower in the first part of the study, especially in patients with isolated aortic valves (47.8 vs 52.7 min, *P* = 0.008), mainly related to the increased portion of patients treated with minimally invasive approaches in the second part of the study. The size of the implanted valves did not differ significantly as a function of the year the valve was implanted (*P* = 0.21). Ballooning of the valve was largely abandoned from January 2016, except for a small number of cases (6.6%, n = 14) with a small aortic annulus (20/21 mm annulus measured in the preoperative echocardiogram and with an S-size Perceval valve implanted) during a brief transitional period ending before January 2017, when it was completely discontinued. Because the number of these patients was very low, a third transitional group of patients was not constituted because it would have the risk of representing a potentially incoherent statistical analysis.

**Table 2:** Perioperative characteristics of the patients before and after 2016

Total patients = 286	75 patients (<2016)	211 patients (>2016)	*P*
	mean (SD)/% (n)	mean (SD)/% (n)	
Sternotomy	53.3% (40)	26% (55)	0.0001
Ministernotomy	8% (6)	38.4% (81)	0.0001
RAMT	38.7% (29)	35.6% (75)	0.67
Bicuspid valve	5.3% (4)	14.7% (31)	0.03
Cross-clamp time	50 (16.6) min	56.8 (17.5) min	0.002
• Isolated AVR	47.8 (16) min	52.7 (15.6) min	0.008
• Combined surgery	66.2 (11.1) min	69.6 (17.1) min	0.57
CPB time	72.3 (23.6) min	78.1 (23.2) min	0.04
• Isolated AVR	69.6 (22.4) min	74.6 (21.6) min	0.07
• Combined surgery	92.1 (22.5) min	89 (24.5) min	0.65
Cardioplegia (cold blood)	100% (75)	99% (209)	0.62
Valve size			0.21
S	20% (15)	11.3% (24)	
M	26.6% (20)	34.1% (72)	
L	36% (27)	33.2% (70)	
XL	17.4% (13)	21.4% (45)	
Balloon	94.6% (71)	6.6% (14)	0.00001
Second cross-clamp	1.3% (1)	0.9% (2)	0.71

AVR: aortic valve replacement; CPB: cardiopulmonary bypass; L: large; M: medium; RAMT: right anterior minithoracotomy; S: small; XL: extra large; SD: standard deviation.

A second clamping of the aorta for valve repositioning was needed in 3 cases (1%); it always became necessary after transoesophageal echocardiographic observations of a Perceval paravalvular leak, following the initial release of the aortic clamp. The same Perceval valve was then repositioned on the aortic annulus of all 3 patients. All patients in this cohort ultimately left the operating room with no prosthetic paravalvular leak.

### Postoperative outcomes

A summary of postoperative observations between the 2 groups is given in Table 3. For the entire cohort, the median extubation time was 3 h [2–5]. It was lower in the second part of the study because early postoperative rehabilitation protocols have become more popular in our centre in recent years. The average stay in the intensive care unit was 2.4 (2.6) days. Blood transfusions were necessary in 46.8% (n = 34) of the patients. Table 3 also gives the rate of severe (< 50,000 x 109/l) postoperative thrombopenia (9% in the entire cohort). It tended to peak on postoperative day 4 and resolve spontaneously shortly thereafter, without leading to appreciable increases in morbidity or mortality. The mean aortic gradient at discharge was lower in the second part of the study (*P* = 0.01) without any clear explication.

**Table 3:** Postoperative outcomes for the cohort before and after 2016

Total patients = 286	75 patients (<2016); mean (SD)/median [range]/% (n)	211 patients (>2016); mean (SD)/median [range]/% (n)	*P*
Extubation time	5 [4-7] hours	2 [2-4] hours	0.0001
Chest drain volume at 12 h	330 [230-460] mL	310 [210-470] mL	0.33
ICU stay	2.1 (2.1) days	2.4 (2.7) days	0.50
New onset postoperative atrial fibrillation	29.3% (22)	27.9% (59)	0.82
New onset PCM	16% (12)	5.6% (12)	0.005
Postoperative severe thrombopenia < 50 000 10^9^/l	10.6% (8)	8.5% (18)	0.64
LVEF at discharge	57 (9) %	57.2 (8.2) %	0.92
Aortic mean gradient at discharge	14.3 (5.2) mmHg	12.5 (3.8) mmHg	0.01
Hospital stay (including ICU stay)	7.8 (2.6) days	8.6 (4.6) days	0.89
Postoperative morbidity	21.3% (16)	25.1% (53)	0.11
Postoperative 30-day mortality	5.3% (4)	4.2% (9)	0.95

ICU: intensive care unit; LVEF: left ventricular ejection fraction; PCM: pacemaker; SD: standard deviation.

Postoperative morbidity from all causes was seen in 24.1% of cases (n = 69). The most frequent complications were lower respiratory infections (n = 18), general sepsis (n = 10) and low output syndrome (n = 9). Bleeding revisions within 24 h postoperatively were performed in 7 cases (2.4%) and occurred at a rate similar to that of patients treated with sternotomy and minimally invasive techniques (*P* = 0.2). Late pericardial effusion after postoperative day 4 was observed in 7 patients (2.4%) and was more frequent in the sternotomy than in the minimally invasive surgery group (5.2% vs 1%, *P* = 0.04). Overall postoperative mortality was 4.5% (n = 13), but only 3.5% (n = 8) among patients who received isolated aortic valve replacements. Two 2 patients died of sudden death (both after postoperative day 15 and with no transient heart block during the stay in our department); 2 patients died of significant strokes; and 9 patients died of other aetiologies (pulmonary embolism, multiorgan failure, aortic rupture at the level of the aortotomy with normal aortic annulus, low output syndrome and mesenteric ischaemia).

### Perceval and postoperative pacemaker rate

**Figure 2:**
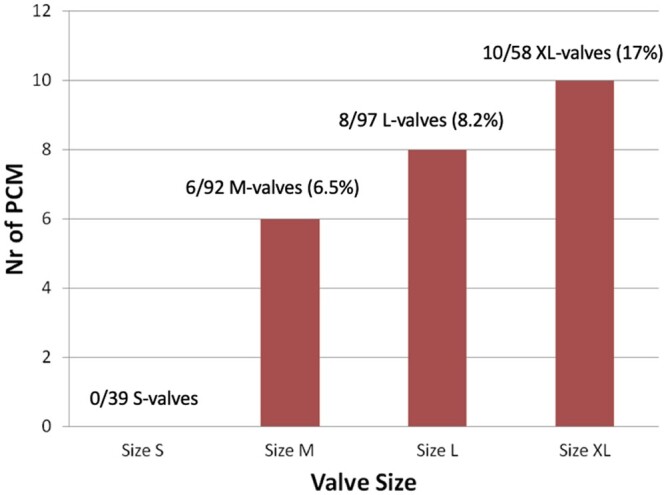
Distribution of the pacemakers according to the size of the valve. Results are presented as bars (absolute numbers) and as percentage of the different sizes of the valves implanted. L: large; M: medium; Nr: number; PCM: permanent cardiac pacemaker; S: small; XL: extra large.

The overall pacemaker rate in the cohort was 8.4% (n = 24), but only 7% (n = 16) in patients with isolated aortic valves. The overall rate decreased significantly after we changed the surgical technique for implanting the Perceval valve in 2016 (16% rate before 2016 vs 5.6% rate after 2016, *P* = 0.005) (Central Figure) with an adjusted odds ratio of 0.31 (95% confidence interval: 0.13–0.74, *P* = 0.012) for the second period of the study compared to the first one. The same trend was also seen in the subgroup of patients with isolated aortic valve replacement (16% rate before 2016 vs 3.1% rate after 2016, Bonferroni correction *P* = 0.001). The mean period of postoperative pacemaker implants was 9.2 (6.4) days, which did not significantly differ before and after 2016 (*P* = 0.51). Fig. 2 shows the distribution of pacemakers according to the size of the valve.

Table 4 shows the univariable and multivariable analyses of the factors chosen as clinically relevant and potentially related to the rate of pacemakers inserted postoperatively. First the univariable and then the multivariable analysis showed that larger valve size (*P* = 0.01) and ballooning (HR = 3.86, *P* = 0.002) were associated with a higher risk of having a pacemaker implanted. The risk increased from HR 1.75 for M-sized valves to HR 5.33 for XL-sized valves.

**Table 4:** Variables potentially influencing postoperative pacemaker rate tested in univariable (each group before and after 2016) and multivariable analyses

Variables	75 patients (<2016)	211 patients (>2016)
	Univariable analysis	Univariable analysis
Age	0.55	0.43
Gender	0.92	0.009
Ischaemic cardiomyopathy	0.83	0.06
Redo surgery	0.70	0.06
Preoperative atrial fibrillation	0.47	0.11
Surgical access	0.44	0.12
(Sterno/RAMT/Ministerno)		
Isolated AVR	0.95	0.09
Bicuspid aortic valve	0.84	0.29
Cross-clamp time	0.55	0.07
Valve size	0.04	0.01
Balloon	0.02	0.7

	Multivariable analysis
Valve size	(*P* = 0.01)
	S size: no pacemaker implanted
	M size: HR 1.75 (95% CI: 1.12-2.72)
	L size: HR 3.05 (95% CI: 1.26-7.38)
	XL size: HR 5.33 (95% CI: 1.42-20.06)
Balloon	HR 3.86 (*P* 0.002) (95% CI: 1.63-9.16)

AVR: aortic valve replacement; CI: confidence interval; HR: hazard ratio; L: large; M: medium; RAMT: right anterior minithoracotomy. RAMT: right anterior minithoracotomy; S: small; XL: extra large;

## DISCUSSION

Early studies on Perceval valve replacement initially reported postoperative pacemaker rates between 4.2 and 6% (7–9). Clinical outcomes began to diverge in articles published between 2016 and 2019. During this period, this technique was being adopted more widely across the world; also during this period, pacemaker rates between 9% and up to 24.1% [10–16] were reported from studies based on clinical results obtained before 2016 (Central Figure), making it necessary to introduce major modifications in the way these interventions were administered.

We sought to address the question of whether postoperative pacemaker rates were constant and inherent in Perceval valve implants, or whether their risk was modifiable and amenable to improvements in surgical protocols. We concluded that lower pacemaker rates can be achieved through 3 different changes in operating techniques.

The first change is to place the Perceval valve in a higher position after total aortic decalcification. Total decalcification, beyond what is usually required for a better sized and well-seated valve, may pre-empt the insertion of the calcified mass through the conductive tissues of the heart. Further improvements can be made with respect to the implant. Passing the 3 guiding sutures 2 mm below the nadir of the cusps, which was applied in earlier procedures, may result in a low circumferential position of the inflow portion of the stent of the Perceval prosthesis and in compression of the conducting tissues of the heart. Inserting the valve in a higher position, with the guiding sutures passed through the annulus, as routinely performed for sutured valves, is likely to reduce the risk of implanting a pacemaker [17]. Implanting the valve in a higher position was initially advised against due to concerns that it could lead to valve displacement or paravalvular leak, concerns that later were found to be unjustified. The valves are designed such that the nitinol stent can easily stabilize at the level of the aortic root and particularly at the sinotubular junction level.

Second, ballooning the valve may increase the rate of pacemaker insertions. We believe that it may cause damage to the conduction tissue, through postoperative irritation or oedema, often leading to temporary postoperative atrioventricular block. Charles Blouin and colleagues [18] found no evidence of an association between ballooning and implanting a pacemaker. Interestingly, their work focused on the relationship between non-ballooning and paravalvular leaks. Their work seems to mirror our experience with nitinol transaortic catheter valves, where ballooning is no longer used systematically but is reserved only for exceptional cases of heavily calcified valves. Total calcium debridement of the annulus may remove the need for Perceval valve ballooning, because the radial force of its nitinol stent may be sufficient to assure valve stability. In our experience, ballooning did not affect the risk of paravalvular leaks, but was an independent predictor of the need to implant a pacemaker.

**Figure 3:**
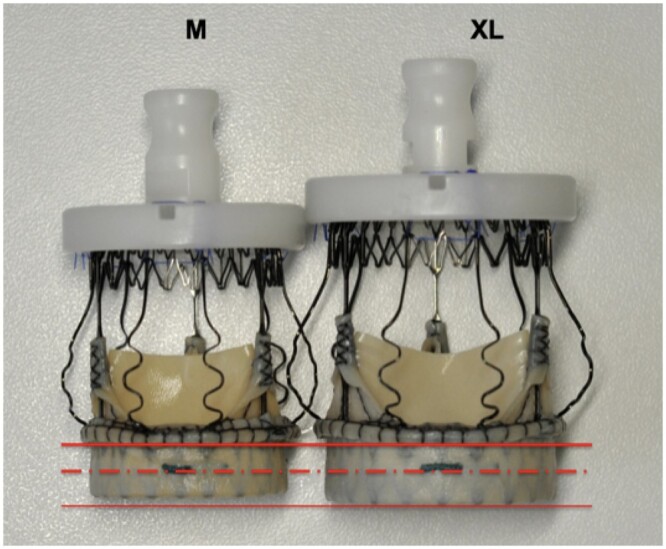
Photograph of a medium-sized and an extra-large-sized Perceval valve. The red lines clearly show that the inflow portion of the stent of the valve is higher in the extra large valve. M: medium; EL: extralarge.

**Figure 4:**
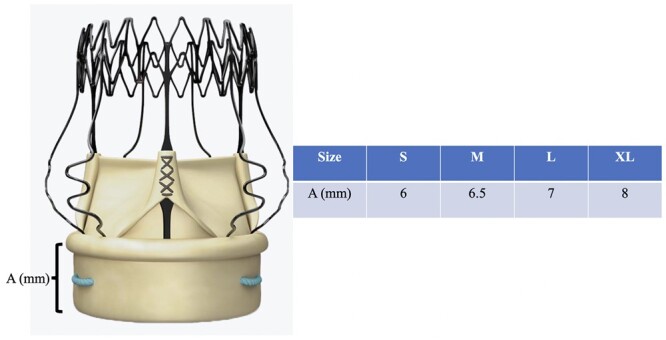
Distances measured of the inflow portion of the stent of the Perceval valve according to the size of the valve (courtesy of CORCYM, previously LivaNova). L: large; M: medium; S: small; XL: extra large.

Third, valve oversizing is a risk factor contributing to the need to implant a pacemaker. Sizing the annulus with the help of a white obturator may be misleading. The choice of the correct valve size is often subjective, being made on the basis of several “gut-feeling” parameters such as visual assessment, tactile feedback, stiffness of the aortic annulus, the space between the obturator and the annulus and calcification of the root, all of which are difficult to quantify objectively. However, 2 technical factors affect the decision about the correct valve size: (i) the final size of the valve is bigger than the corresponding white obturator (Fig. 1) and the length of the inflow portion of the stent of the Perceval increases proportionally with the size of the valve (Figs. 3, 4). Oversized valves increase the risk of the need for a pacemaker [19, 20] (Fig. 2). This result was also supported by the findings of the PERSIST trial [4]; almost half of the pacemakers were observed after the XL size valves were implanted. The oversizing of the valve can significantly stretch the aortic annulus and lead to temporary or permanent postoperative atrioventricular conduction issues. In borderline cases, we recommend smaller valve sizes. The radial force of the nitinol stent alone may also play a role at the annular level, and significant valve downsizing did not appear to lead to increased rates of paravalvular leaks in our patients.

The presence of pre-existing atrio- or intraventricular conduction anomalies also deserves particular attention. We found no association between these 2 types of conduction anomalies and the rate of pacemakers inserted postoperatively (data not shown), but Brookes and colleagues [21] suggested that preoperative right bundle branch block, prolonged QRS complex and longer PR intervals are associated with an increased risk of the need to insert a pacemaker postoperatively. Therefore, the presence of conduction abnormalities preoperatively should be evaluated when one is contemplating implanting a Perceval valve in these patients.

Lastly, those responsible for the recent PERSIST trial [4] should be commended for introducing prospective randomized methods in cardiac surgery, particularly in the valvular field. Nevertheless, several questions remain, notably, the risk of needing a pacemaker postoperatively, which was the focus of our study. The PERSIST trial, which covered an enrollment period between 2017 and 2019, found a rate as high as 10.6% at 30 days (vs 3.2% in the sutured valve group). After 2016, in our centre, we only observed an overall 2.7% pacemaker implant rate among patients with sutured aortic valves (isolated aortic valve replacement or combined surgery) and an overall rate of pacemakers of 5.6% among all patients with Perceval valves. This rate was even lower (3.1%) among patients with isolated aortic replacement Perceval valves.

Our study has the limitations inherent in its retrospective single-centre study design. The sequential aspect over time of the technical changes related to surgical implants may have resulted in a cohort that was not homogeneous. Moreover, during 2016, a transition period occurred during which 14 patients still underwent ballooning of the valve. Also, these results were based on the work of 2 surgeons and therefore may not be easily generalizable across other, potentially larger centres. In addition, the decision to implant a pacemaker postoperatively is made locally by cardiologists, which further increases the heterogeneity of the outcomes. This study describes results obtained after concomitant changes in 2 operative elements, so it is difficult to evaluate the influence of any individual element. Although ballooning was a clear risk factor, the higher implant location and the absence of oversizing of the valve, may also have played a role in the decrease of the number of pacemakers inserted postoperatively.

## CONCLUSION

Our findings are consistent with those of other recent reports [3, 22], suggesting that the rate of pacemakers implanted after the Perceval implant may be reduced by improving the implant procedure. In isolated patients with aortic valve issues, the pacemaker rate should be sufficiently reduced to become comparable with that following sutured valve procedures. Other centres, where implanting Perceval valves may be a new endeavour, may want to use our experience to their advantage and avoid the pitfalls that were present during the early adoption stage elsewhere. Adopting best practices will allow a wider use of minimally invasive approaches, especially a right anterior minithoracotomy, in which the benefits of Perceval valve implants are greatest. Whether valve profile improvements, which comprise a shortening of the inflow portion of the stent of the valve made by the manufacturer (Perceval Plus valve), may have added benefits needs to be evaluated and confirmed through additional research.

## Funding

No funding was provided for this research project.

**Conflicts of interest:** Dr Fabre is a consultant for CORCYM, previously LivaNova (Proctor). The other authors have no additional conflicts of interest to declare.

## Data availability

All relevant data are within the manuscript and its supporting information files.

## References

[1] Shrestha M , Khaladj N, Bara C, Hoeffler K, Hagl C, Haverich A. A staged approach towards interventional aortic valve implantation with a sutureless valve: initial human implants. Thorac Cardiovasc Surg oct 2008;56:398–400. 18810696 10.1055/s-2008-1038722

[2] Shrestha M , Folliguet T, Meuris B, Dibie A, Bara C, Herregods M-C et al Sutureless Perceval S aortic valve replacement: a multicenter, prospective pilot trial. J Heart Valve Dis nov 2009;18:698–702. 20099720

[3] Glauber M , Di Bacco L, Cuenca J, Di Bartolomeo R, Baghai M, Zakova D et al Minimally Invasive Aortic Valve Replacement with Sutureless Valves: results From an International Prospective Registry. Innovations (Phila) avr 2020;15:120–30. 31875777 10.1177/1556984519892585

[4] Fischlein T , Folliguet T, Meuris B, Shrestha ML, Roselli EE, McGlothlin A et al Sutureless versus conventional bioprostheses for aortic valve replacement in severe symptomatic aortic valve stenosis. J Thorac Cardiovasc Surg mars 2021;161:920–32. 33478837 10.1016/j.jtcvs.2020.11.162

[5] Fabre O , Carjaliu I, Rebet O, Radutoiu M, Gautier L, Durand F et al Reversed C-shaped scanner-guided ministernotomy for isolated aortic valve replacement. Ann Thorac Surg. 17 oct 2020; 10.1016/j.athoracsur.2020.07.076 33080238

[6] Hysi I , Rebet O, Carjaliu I, Radutoiu M, Gautier L, Fabre O, Right anterior minithoracotomy with total central cannulation. Multimed Man Cardiothorac Surg MMCTS12 févr 2021;2021. 10.1510/mmcts.2021.006 33645931

[7] Santarpino G , Pfeiffer S, Schmidt J, Concistrè G, Fischlein T. Sutureless aortic valve replacement: first-year single-center experience. Ann Thorac Surg août 2012;94:504–8. discussion 508–509. 22695049 10.1016/j.athoracsur.2012.04.024

[8] Miceli A , Santarpino G, Pfeiffer S, Murzi M, Gilmanov D, Concistré G et al Minimally invasive aortic valve replacement with Perceval S sutureless valve: early outcomes and one-year survival from two European centers. J Thorac Cardiovasc Surg déc 2014;148:2838–43. 24698558 10.1016/j.jtcvs.2014.02.085

[9] Zannis K , Joffre J, Czitrom D, Folliguet T, Noghin M, Lansac MNE et al Aortic valve replacement with the perceval S bioprosthesis: single-center experience in 143 patients. J Heart Valve Dis nov 2014;23:795–802. 25790630

[10] Vogt F , Pfeiffer S, Dell'Aquila AM, Fischlein T, Santarpino G. Sutureless aortic valve replacement with Perceval bioprosthesis: are there predicting factors for postoperative pacemaker implantation? Interact CardioVasc Thorac Surg mars 2016;22:253–8. 26614526 10.1093/icvts/ivv330 PMC4986555

[11] Toledano B , Bisbal F, Camara ML, Labata C, Berastegui E, Gálvez-Montón C et al Incidence and predictors of new-onset atrioventricular block requiring pacemaker implantation after sutureless aortic valve replacement. Interact CardioVasc Thorac Surg déc 2016;23:861–8. 27572616 10.1093/icvts/ivw259

[12] Bouhout I , Mazine A, Rivard L, Ghoneim A, El-Hamamsy I, Lamarche Y et al Conduction Disorders After Sutureless Aortic Valve Replacement. Ann Thorac Surg avr 2017;103:1254–60. 27717423 10.1016/j.athoracsur.2016.07.044

[13] Liakopoulos OJ , Gerfer S, Weider S, Rahmanian P, Zeriouh M, Eghbalzadeh K et al Direct Comparison of the Edwards Intuity Elite and Sorin Perceval S Rapid Deployment Aortic Valves. Ann Thorac Surg janv 2018;105:108–14. 29042007 10.1016/j.athoracsur.2017.06.034

[14] Berretta P , Andreas M, Carrel TP, Solinas M, Teoh K, Fischlein T et al Minimally invasive aortic valve replacement with sutureless and rapid deployment valves: a report from an international registry (Sutureless and Rapid Deployment International Registry)†. Eur J Cardiothorac Surg 1 oct 2019;56:793–9. 30820549 10.1093/ejcts/ezz055

[15] Suri RM , Javadikasgari H, Heimansohn DA, Weissman NJ, Ailawadi G, Ad N et al Prospective US investigational device exemption trial of a sutureless aortic bioprosthesis: one-year outcomes. J Thorac Cardiovasc Surg mai 2019;157:1773–1782.e3. 30553598 10.1016/j.jtcvs.2018.08.121

[16] Mugnai G , Moran D, Nijs J, Chierchia G-B, Velagic V, Ströker E et al Electrocardiographic and clinical predictors of permanent pacemaker insertion following Perceval sutureless aortic valve implantation. J Electrocardiol oct 2019;56:10–4. 31229677 10.1016/j.jelectrocard.2019.06.004

[17] Mashhour A , Zhigalov K, Mkalaluh S, Szczechowicz M, Easo J, Eichstaedt HC et al Outcome of a Modified Perceval Implantation Technique. Thorac Cardiovasc Surg oct 2020;68:602–7. 31003238 10.1055/s-0039-1685512

[18] Charles Blouin M , Bouhout I, Demers P, Carrier M, Perrault L, Lamarche Y et al Tackling the Issue of High Postoperative Pacemaker Implantation Rates in Sutureless Aortic Valve Replacement: should Balloon Inflation be Removed from the Implantation Method of the Perceval Prosthesis? J Heart Valve Dis mai 2017;26:247–54. 29092107

[19] González Barbeito M , Estévez-Cid F, Pardo Martínez P, Velasco García de Sierra C, Iglesias Gil C, Quiñones Laguillo C et al Surgical technique modifies the postoperative atrioventricular block rate in sutureless prostheses. J Thorac Dis juill 2019;11:2945–54. 31463124 10.21037/jtd.2019.07.27 PMC6687973

[20] Geršak B , Glauber M, Bouchard D, Jug J, Solinas M. Oversizing Increases Pacemaker Implantation Rate After Sutureless Minimally Invasive Aortic Valve Replacement. Innov Phila Pa oct 2020;15:449–55. 10.1177/1556984520938897 32758051

[21] Brookes JDL , Mathew M, Brookes EM, Jaya JS, Almeida AA, Smith JA. Predictors of Pacemaker Insertion Post-Sutureless (Perceval) Aortic Valve Implantation. Heart Lung Circ 2021;30(6):917–921. 10.1016/j.hlc.2020.11.004 33309876

[22] D'Onofrio A , Salizzoni S, Filippini C, Tessari C, Bagozzi L, Messina A et al Surgical aortic valve replacement with new-generation bioprostheses: sutureless versus rapid-deployment. J Thorac Cardiovasc Surg févr 2020;159:432–442.e1. 31213376 10.1016/j.jtcvs.2019.02.135

